# Vasopressin Versus Norepinephrine As First-Line Vasopressor Therapy for Vasoplegic Shock Following Cardiac Surgery: A Systematic Review

**DOI:** 10.7759/cureus.112528

**Published:** 2026-07-12

**Authors:** Aabha Divya, Niti Dalal, Grainne McKendry, Morgan Quinn, Aranya Bagchi, Adam Dalia, Kenneth Shelton

**Affiliations:** 1 Department of Cardiothoracic Surgery, Tulane University School of Medicine, New Orleans, USA; 2 Department of Cardiac Surgery and Cardiac Intensive Care Unit, Royal Victoria Hospital, Belfast, Belfast, IRL; 3 Department of Anaesthesia, Flinders University, Adelaide, AUS; 4 Department of Anaesthesiology, Massachusetts General Hospital, Boston, USA

**Keywords:** cardiac surgery, cardiopulmonary bypass, norepinephrine, vasoplegic shock, vasopressin

## Abstract

Vasoplegic shock following cardiac surgery is associated with significant morbidity. While norepinephrine is recommended as first-line therapy, vasopressin has been proposed as an alternative due to catecholamine-independent mechanisms. Comparative evidence in postcardiac surgery populations remains limited. We conducted a systematic review of randomized and observational studies comparing vasopressin versus norepinephrine as initial vasopressor therapy in adults with vasoplegic shock following cardiac surgery with cardiopulmonary bypass. Primary outcomes included mortality and major postoperative complications. Risk of bias was assessed using the Cochrane Risk of Bias 2 (RoB 2) and the Risk of Bias in Non-randomized Studies of Interventions (ROBINS-I) tools. Two studies (n = 638 patients) met inclusion criteria: one randomized trial (VANCS) and one propensity score-matched observational cohort study. In the randomized trial, vasopressin was associated with a lower incidence of composite morbidity (32.2% vs 49.0%), reduced incidence of acute kidney injury requiring dialysis, and shorter ICU stay, without a difference in mortality. In contrast, the observational study in patients with severe left ventricular (LV) dysfunction showed no benefit and suggested higher rates of arrhythmias and mortality with vasopressin. Available evidence suggests that the effect of vasopressin in postcardiac surgery vasoplegia may depend on baseline ventricular function. Vasopressin may be considered as an early catecholamine-sparing strategy in selected patients with preserved ventricular function, but current evidence does not support a universal change from norepinephrine to vasopressin as first-line therapy for all patients. In patients with severe ventricular dysfunction, vasopressin should be used cautiously, and adequately powered phenotype-stratified randomized trials are needed.

## Introduction and background

Vasoplegic shock is a well-recognized complication after cardiac surgery with cardiopulmonary bypass (CPB), characterized by severe hypotension, low systemic vascular resistance, and preserved or increased cardiac output despite adequate volume resuscitation [1,2]. The reported incidence of vasoplegic shock following cardiac surgery with CPB ranges from 5% to 25%, depending on the population studied and diagnostic criteria applied, and has been associated with increased organ dysfunction, prolonged intensive care unit (ICU) stay, and higher healthcare costs [1,3]. Vasoplegia is thought to result from a CPB-triggered inflammatory response, involving excessive nitric oxide production, activation of inflammatory mediators, dysregulated vasopressor signaling, and endothelial dysfunction [3,4].

Norepinephrine is widely recommended as the first-line vasopressor for vasodilatory shock, which includes the vasoplegic state after cardiac surgery [5]. In this review, we use the term vasodilatory shock to describe pathologic loss of vascular tone across clinical contexts, whereas vasoplegic shock refers specifically to the post-CPB syndrome characterized by hypotension, low systemic vascular resistance, and preserved or increased cardiac output despite adequate volume resuscitation. Accordingly, this review focuses not on routine transient hypotension after CPB, but on established post-CPB vasoplegic shock requiring vasopressor therapy.

Vasoplegia may also involve relative arginine vasopressin deficiency, as described in both septic and postoperative vasodilatory shock, where endogenous vasopressin levels initially rise but subsequently fall as stores are depleted, potentially contributing to persistent vasodilation and catecholamine resistance [4,6]. This rationale has generated interest in vasopressin as an alternative or adjunct vasopressor in vasoplegic shock. Although general shock guidelines favor norepinephrine as first-line therapy for vasodilatory shock, guidance after cardiac surgery is less clear, typically endorsing norepinephrine and/or vasopressin to restore perfusion pressure. However, available guidance does not clearly define whether vasopressin should be preferred as initial therapy in specific postcardiac surgery phenotypes, particularly according to baseline ventricular function [5]. Post-cardiac surgery vasoplegic shock is a specific post-CPB phenotype within the broader spectrum of vasodilatory shock. Although vasopressin data from septic and other vasodilatory shock states provide biologic rationale, these findings are indirect and may not fully apply after cardiac surgery, where ventricular reserve and perioperative myocardial dysfunction may influence vasopressor response.

However, the available clinical evidence remains limited and inconsistent. Several small randomized and observational studies suggest that vasopressin may reduce catecholamine requirements and improve hemodynamics in postcardiac surgery vasoplegia. However, these studies differ substantially in patient populations, vasopressor strategies, and reported outcomes, making interpretation of the evidence challenging [7,8]. To our knowledge, no systematic synthesis has specifically evaluated vasopressin versus norepinephrine as the initial vasopressor for vasoplegic shock after cardiac surgery.

We therefore conducted a systematic review, based on a prospectively registered International Prospective Register of Systematic Reviews (PROSPERO) protocol, to evaluate the comparative effectiveness and safety of vasopressin versus norepinephrine as initial vasopressor therapy in adult patients with vasoplegic shock after cardiac surgery with CPB [9]. Our objectives were to summarize all available comparative evidence, assess the overall certainty of evidence for key clinical outcomes, and explore whether baseline left ventricular function may explain heterogeneity in treatment effects.

## Review

Methods

Study Design and Protocol Registration

This systematic review was conducted in accordance with the Preferred Reporting Items for Systematic Reviews and Meta-Analyses (PRISMA 2020) reporting guidelines [9], and the PRISMA checklist is provided in Appendix A. The review protocol was prospectively registered in PROSPERO (CRD420261341741). The objective of this review was to evaluate the comparative effectiveness and safety of vasopressin versus norepinephrine as the initial vasopressor therapy in adult patients with vasoplegic shock following cardiac surgery with CPB. The complete database-specific search strategies, including search terms, Boolean operators, and date of search, are provided in Appendix B.

Eligibility Criteria

Studies were eligible if they met the following criteria: (1) adult patients (defined as age ≥18 years) undergoing cardiac surgery with CPB who developed postoperative vasoplegic shock; (2) vasopressin used as the primary vasopressor therapy; (3) norepinephrine as the comparator; (4) randomized controlled trials or observational comparative studies; and (5) reporting clinical outcomes which included all-cause mortality, need for renal replacement therapy or documented acute kidney injury, arrhythmias, duration of mechanical ventilation, or length of ICU and/or hospital stay. For this review, postoperative vasoplegic shock was defined as hypotension after CPB with low systemic vascular resistance despite adequate volume resuscitation, with preserved or increased cardiac output and a requirement for vasopressor therapy. Because diagnostic criteria for vasoplegia vary across the literature, studies were eligible only when the population was explicitly described as having vasoplegic shock or vasoplegic syndrome after cardiac surgery with CPB.

Studies were excluded if they evaluated routine transient postoperative hypotension, vasopressin prophylaxis rather than treatment of established vasoplegic shock, vasopressin as rescue therapy without a norepinephrine comparator, non-cardiac surgery vasodilatory shock populations, pediatric populations, or if they were non-comparative studies, case reports, reviews, editorials, or conference abstracts without full data.

Outcomes

The primary outcome of interest was the composite postoperative morbidity endpoint as defined by each included study. Secondary outcomes of interest included all-cause 30-day mortality, acute kidney injury, need for renal replacement therapy, new-onset atrial fibrillation, ventricular arrhythmias, cardiac reoperation, extracorporeal membrane oxygenation (ECMO) use, stroke, duration of mechanical ventilation, ICU length of stay, and hospital length of stay. Because composite endpoint definitions differed between included studies, individual component outcomes were extracted and reported separately to facilitate direct comparison across studies.

Literature Search

A systematic search of MEDLINE (PubMed), Embase (Ovid), CENTRAL, Web of Science, and Scopus was conducted from inception through March 2026, with no date or language restrictions. Search strategies combined controlled vocabulary (Medical Subject Headings (MeSH) and Emtree) and free-text keywords for vasopressin, norepinephrine, vasoplegic shock, and cardiac surgery with CPB (Appendix B). Broader terms, including vasodilatory shock and distributive shock, were included to maximize search sensitivity; however, study eligibility required a population of postcardiac surgery, CPB-associated vasoplegic shock. ClinicalTrials.gov and the World Health Organization (WHO) International Clinical Trials Registry Platform (ICTRP) were searched for unpublished and ongoing studies. Additional studies were identified through backward and forward citation searching of included studies and relevant reviews.

Study Selection

Two reviewers independently screened titles and abstracts, followed by full-text articles, against the predefined inclusion and exclusion criteria. Disagreements were resolved through discussion and consensus, with a third reviewer available for adjudication where necessary. Screening was performed using Covidence systematic review software (Veritas Health Innovation, Melbourne, VIC, Australia). The most common reasons for exclusion were evaluation of vasopressin as prophylaxis or adjunctive/rescue therapy, absence of a direct norepinephrine comparator, non-comparative study design, or inclusion of mixed vasodilatory shock populations rather than established postcardiac surgery vasoplegic shock.

Data Extraction

Data were extracted independently by two reviewers using a standardized data extraction form. Extracted variables included study design, country, patient characteristics, sample size, intervention and comparator details, and reported clinical outcomes.

Risk of Bias Assessment

The methodological quality of included studies was assessed using validated tools. The Cochrane Risk of Bias 2 (RoB 2) tool [10] was used for randomized controlled trials, while the Risk Of Bias In Non-randomized Studies of Interventions (ROBINS-I) tool [11] was used for observational studies.

Data Synthesis

Due to the limited number of eligible studies and differences in study design, baseline ventricular function, patient populations, and outcome definitions, quantitative meta-analysis was not performed. Instead, a qualitative narrative synthesis was conducted. Findings from the narrative synthesis were interpreted with caution because pooled effect estimates could not be generated.

Results

Study Selection

**Figure 1:**
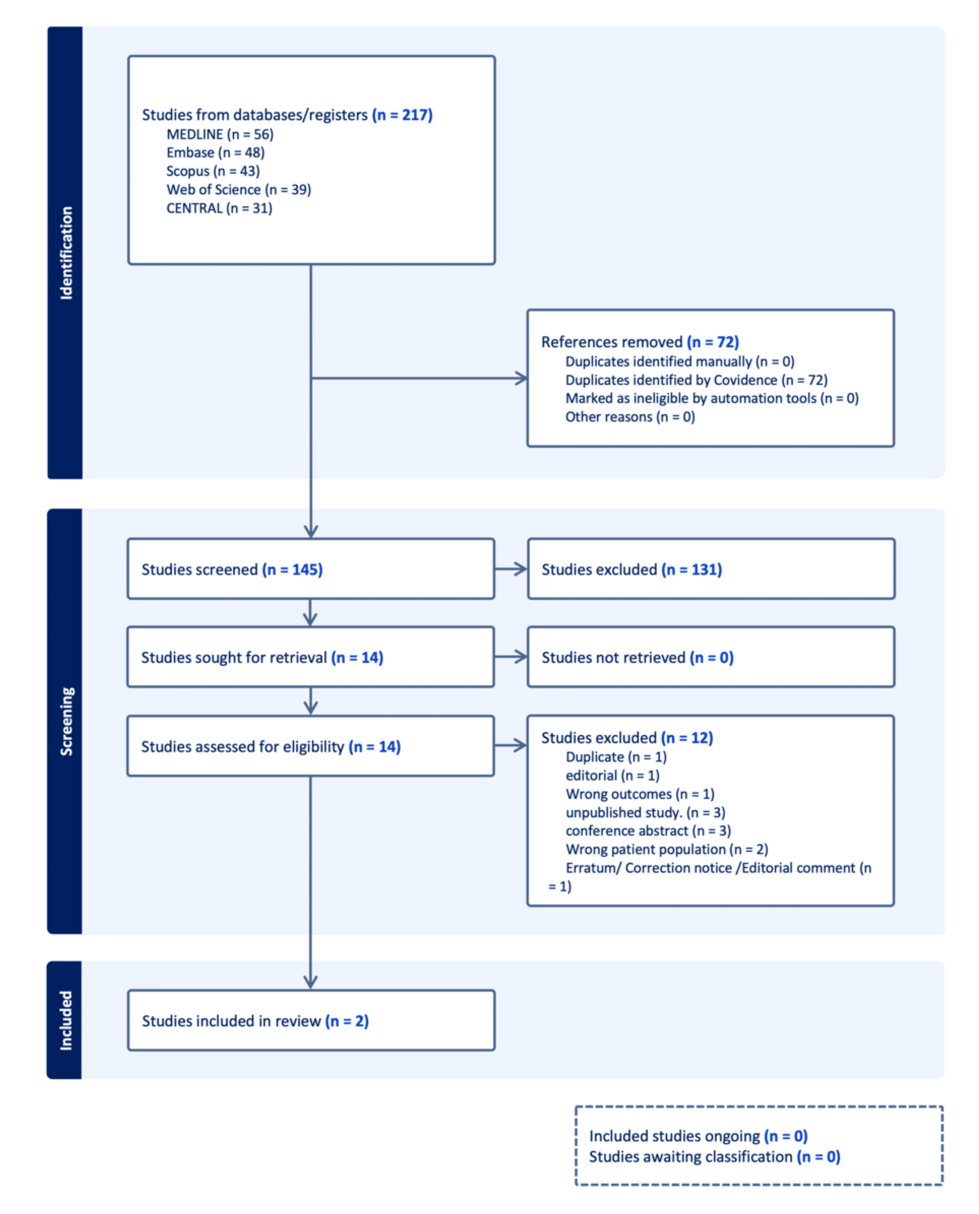
PRISMA Flow Diagram of the Study Selection Process

The literature search identified 217 records across all databases. After 72 duplicates, 145 studies underwent title and abstract screening. Fourteen full-text articles were assessed for eligibility. Twelve studies were excluded due to duplicate publication, editorial format, incorrect outcomes, unpublished data, conference abstracts without full results, or wrong patient population. Ultimately, two studies met the inclusion criteria, including one randomized controlled trial and one retrospective propensity score-matched observational cohort study, comprising a total of 638 patients (Figure 1). The most common reasons for exclusion were evaluation of vasopressin as prophylaxis or adjunctive/rescue therapy, absence of a direct norepinephrine comparator, non-comparative study design, or inclusion of mixed vasodilatory shock populations rather than established postcardiac surgery vasoplegic shock.

Study Characteristics

The randomized VANCS trial [8] evaluated vasopressin compared with norepinephrine as the initial vasopressor therapy in patients who developed vasoplegic shock following cardiac surgery with CPB. Vasopressin was administered at 0.01-0.06 U/min, while norepinephrine doses ranged from 10 to 60 μg/min. The majority of patients had preserved ventricular function, with approximately 82% demonstrating a left ventricular ejection fraction greater than 40%.

The observational cohort study by Cheng et al. [12] included patients with severe preoperative left ventricular dysfunction characterized by markedly reduced ejection fraction and advanced heart failure. Vasopressin was administered at 0.02-0.07 U/min and compared with norepinephrine within a similar dosing range (Table 1). Definitions of vasoplegic shock varied between studies. VANCS required hypotension with low systemic vascular resistance despite adequate fluid resuscitation and preserved cardiac index, consistent with a hemodynamically defined vasoplegic phenotype. In contrast, the observational cohort relied on clinically documented vasoplegic shock after cardiac surgery, and the extent to which monitoring of systemic vascular resistance or cardiac output was applied uniformly was less clear.

**Table 1:** Characteristics of Included Studies *LV dysfunction is defined as LVEF ≤35%. Vasopressor doses are reported as presented in the original studies. CPB: cardiopulmonary bypass; LVEF: left ventricular ejection fraction; LV: left ventricle; AKI: acute kidney injury; COPD: chronic obstructive pulmonary disease

Characteristic	Hajjar et al., 2017 (VANCS) [8]	Cheng et al., 2018 [12]
Design	Randomized double-blind trial	Retrospective propensity score-matched cohort
Country	Brazil (single-center randomized trial)	China (two-center cohort study)
Population	Adults with vasoplegic shock after cardiac surgery with CPB	Adults with severe preoperative LV dysfunction* and vasoplegic shock after cardiac surgery
Intervention	Vasopressin (0.01-0.06 U/min)	Vasopressin (0.02-0.07 U/min)
Comparator	Norepinephrine (10-60 µg/min)	Norepinephrine (10-60 µg/min)
Sample Size	300 patients (149 vasopressin, 151 norepinephrine)	338 propensity score-matched patients (169 vasopressin, 169 norepinephrine)
Key Outcomes	Composite morbidity, AKI, atrial fibrillation, ICU stay, mortality	Composite outcome, AKI, arrhythmias, ICU stay, mortality
Baseline LVEF	~82% with LVEF >40%	100% with LVEF ≤35%
COPD	39.7%	0% (excluded)
Follow-up	30 days	30 days

The composite primary endpoint differed between studies. In the VANCS trial, the composite endpoint consisted of 30-day mortality, acute kidney injury requiring dialysis, prolonged mechanical ventilation >48 hours, stroke, and deep sternal wound infection. In the Cheng et al. cohort, the composite endpoint included 30-day mortality, mechanical ventilation >48 hours, cardiac reoperation, ECMO use, stroke, and severe acute kidney injury defined as the Acute Kidney Injury Network (AKIN) stage II/III. Atrial fibrillation and ventricular arrhythmias were reported as secondary outcomes in the Cheng et al. study, but were included among the postoperative outcomes reported in the VANCS trial.

Primary Outcomes

In the VANCS randomized trial, the composite primary outcome occurred less frequently with vasopressin than with norepinephrine (32.2% vs 49.0%; adjusted HR, 0.52; 95% CI, 0.36-0.75; p = 0.0005). In contrast, the Cheng cohort study reported no statistically significant difference in the composite outcome between treatment groups (58.6% vs 50.9%; p = 0.155).

Renal outcomes showed discordant findings between studies, although outcome definitions differed. In the VANCS randomized trial, acute kidney injury requiring dialysis occurred less frequently among patients treated with vasopressin compared with norepinephrine (10.1% vs 35.8%; adjusted HR 0.26, 95% CI 0.15-0.46; p < 0.0001). Conversely, the Cheng observational study reported similar rates of severe acute kidney injury, defined as AKIN stage II/III, between treatment groups (48.5% vs 43.2%; p = 0.326).

Findings for postoperative atrial fibrillation were similarly discordant across studies, as it occurred less frequently with vasopressin in the randomized trial (63.8% vs 82.1%; adjusted OR 0.37, 95% CI 0.22-0.64; p = 0.0004). However, the observational cohort reported higher rates of atrial fibrillation in the vasopressin group (20.1% vs 11.8%; p = 0.038) (Table 2).

**Table 2:** Primary Outcomes: Composite and Component Outcomes Note: Effect estimates are reported as presented in the original studies. Renal outcome definitions differed between studies. In VANCS [8], the renal outcome was acute kidney injury requiring dialysis. In Cheng et al. [12], the renal outcome was severe acute kidney injury, defined as AKIN stage II/III. VP: vasopressin; NE: norepinephrine; HR: hazard ratio; OR: odds ratio; CI: confidence interval; AKIN: Acute Kidney Injury Network

Outcome	Hajjar et al., 2017 VP [8] (n=149)	Hajjar et al., 2017 NE [8] (n=151)	Effect Estimate (95% CI)	p value	Cheng et al., 2018 VP [12] (n=169)	Cheng et al., 2018 NE [12] (n=169)	p-value
Composite primary outcome	48 (32.2%)	74 (49.0%)	Adj. HR 0.52 (0.36-0.75)	0.0005	99 (58.6%)	86 (50.9%)	0.155
Renal outcomes	15 (10.1%)	54 (35.8%)	Adj. HR 0.26 (0.15-0.46)	<0.0001	82 (48.5%)	73 (43.2%)	0.326
Atrial fibrillation	95 (63.8%)	124 (82.1%)	Adj. OR 0.37 (0.22-0.64)	0.0004	34 (20.1%)	20 (11.8%)	0.038

Secondary Outcomes

Mortality outcomes differed between studies. Thirty-day mortality did not differ between treatment groups in the randomized trial (15.4% vs 15.9%; adjusted HR 1.11, 95% CI 0.62-1.96; p = 0.73). In contrast, the observational study reported higher mortality among patients receiving vasopressin compared with norepinephrine (5.9% vs 1.8%; p = 0.047).

In the randomized trial, renal replacement therapy occurred less frequently in the vasopressin group (2.7% vs 13.9%; OR 0.17, 95% CI 0.06-0.51; p = 0.0016). This outcome was not reported in the observational study.

Rates of prolonged mechanical ventilation (>48 hours) were similar between treatment groups in both studies (5.4% vs 8.6%; p = 0.30 in the randomized trial and 21.3% vs 22.5%; p = 0.574 in the observational study). Ventricular arrhythmias occurred at comparable rates in the randomized trial (18.1% vs 21.2%; p = 0.45), whereas the observational study reported higher rates in the vasopressin group (24.9% vs 14.2%; p = 0.014).

ICU length of stay demonstrated opposing trends between studies. In the randomized trial, median ICU length of stay was shorter among patients receiving vasopressin compared with norepinephrine (5 (4-7) vs 6 (4-9) days; p = 0.007). In contrast, the observational cohort reported a longer ICU stay in the vasopressin group (6 (4-7) vs 5 (3-6) days; p = 0.002). Similarly, hospital length of stay favored vasopressin in the randomized trial (10 (8-12) vs 13 (10-20) days; p = 0.002), whereas no significant difference was observed in the observational study (23 (21-29) vs 24 (18-29) days; p = 0.185).

In the observational study, the duration of mechanical ventilation was longer among patients treated with vasopressin (23 (16-40) vs 16 (11-23) hours; p = 0.001) (Table 3).

**Table 3:** Secondary Outcomes Notes: Effect estimates represent adjusted analyses as reported in the original studies. Renal replacement therapy was reported separately in VANCS [8] but was not reported in the Cheng et al. cohort [12]. * indicates a statistically significant difference between groups. VP: vasopressin; NE: norepinephrine; HR: hazard ratio; OR: odds ratio; CI: confidence interval; MD: mean difference; IQR: interquartile range

Outcome	Hajjar et al., 2017 VP [8] (n = 149)	Hajjar et al., 2017 NE [8] (n = 151)	Effect Estimate (95% CI)	p-value	Cheng et al., 2018 VP [12] (n = 169)	Cheng et al., 2018 NE [12] (n = 169)	p-value
30-day mortality	23 (15.4%)	24 (15.9%)	Adj. HR 1.11 (0.62-1.96)	0.73	10 (5.9%)	3 (1.8%)	0.047*
Renal replacement therapy	4 (2.7%)	21 (13.9%)	OR 0.17 (0.06-0.51)	0.0016	Not reported	Not reported	-
Mechanical ventilation >48 hours	8 (5.4%)	13 (8.6%)	Adj. OR 0.62 (0.26-1.51)	0.30	36 (21.3%)	38 (22.5%)	0.574
Ventricular arrhythmias	27 (18.1%)	32 (21.2%)	Adj. OR 0.80 (0.45-1.43)	0.45	42 (24.9%)	24 (14.2%)	0.014*
Stroke	4 (2.7%)	4 (2.6%)	Adj. OR 1.08 (0.27-4.39)	0.91	3 (1.8%)	6 (3.6%)	0.311
ICU length of stay (days, median (IQR))	5 (4-7)	6 (4-9)	MD -2.28 (-3.94 to -0.62)	0.007	6 (4-7)	5 (3-6)	0.002*
Hospital length of stay (days, median (IQR))	10 (8-12)	13 (10-20)	MD -3.66 (-6.01 to -1.32)	0.002	23 (21-29)	24 (18-29)	0.185
Duration of mechanical ventilation (hours, median (IQR))	Not reported	Not reported	-	-	23 (16-40)	16 (11-23)	0.001*

Risk of Bias

Using the RoB 2 tool, the randomized VANCS trial by Hajjar et al. was classified as having some concerns, primarily related to potential issues in outcome reporting despite otherwise appropriate randomization and blinding. The observational study by Cheng et al. was assessed using the ROBINS-I tool and classified as having a serious risk of bias due to residual confounding inherent in retrospective analyses, despite propensity score matching (Appendix C). The certainty of evidence for key outcomes was summarized using the Grading of Recommendations Assessment, Development and Evaluation (GRADE) (Appendix D), with the domain-level GRADE assessment provided in Appendix E.

Discussion

Vasoplegic shock remains a clinically significant complication following cardiac surgery with CPB and is associated with increased morbidity, prolonged ICU stay, and higher healthcare utilization [1-3]. In this systematic review, we identified only two comparative studies evaluating vasopressin versus norepinephrine as initial therapy for established postcardiac surgery vasoplegia, highlighting a major evidence gap. Most available studies have evaluated vasopressin as prophylaxis, adjunctive therapy, or rescue therapy rather than as an initial vasopressor strategy. In addition, definitions of postcardiac surgery vasoplegia vary across studies, and many reports include mixed vasodilatory shock populations or emphasize short-term hemodynamic and catecholamine-sparing endpoints rather than clinically meaningful postoperative outcomes. Broader reviews of vasopressin-containing strategies in distributive shock and perioperative patients provide useful context, but they include heterogeneous populations and intervention strategies; therefore, they were not directly applicable to the focused question of whether vasopressin or norepinephrine is superior as initial therapy for established postcardiac surgery vasoplegic shock [13,14]. The divergent findings of the two included studies suggest that treatment response may be modified by baseline ventricular function and support the need for phenotype-specific approaches to vasopressor selection after cardiac surgery.

Pathophysiological Rationale

The pathophysiology of vasoplegic shock after CPB is multifactorial and involves systemic inflammatory activation, endothelial dysfunction, nitric oxide overproduction, and dysregulation of vascular tone [2-4,15]. CPB triggers the release of inflammatory mediators and nitric oxide, leading to profound vasodilation and impaired responsiveness to catecholamines [3,15]. In addition to inflammatory mechanisms, studies have demonstrated that patients with vasodilatory shock may develop a relative deficiency of arginine vasopressin during the later stages of shock [4,16]. Circulating vasopressin concentrations initially increase in response to hypotension but may subsequently decline as endogenous stores are depleted, potentially contributing to persistent vasodilation and catecholamine resistance. This physiological mechanism provides a strong rationale for the therapeutic use of exogenous vasopressin in vasoplegic states.

Current consensus guidelines for circulatory shock recommend norepinephrine as the first-line vasopressor because of its potent α-adrenergic vasoconstrictive effects and favorable safety profile [5]. However, vasopressin acts through non-adrenergic V1 receptors and can restore vascular tone independently of catecholamine signaling pathways. This pharmacologic mechanism may be particularly advantageous in patients with catecholamine-refractory vasodilation. Excessive catecholamine exposure has also been associated with adverse cardiovascular effects, including arrhythmias and increased myocardial oxygen demand, providing additional rationale for catecholamine-sparing vasopressor strategies [17]. Early clinical studies and randomized trials have demonstrated that low-dose vasopressin infusion can improve hemodynamics and reduce catecholamine requirements in vasodilatory shock states [16,18,19].

Although vasopressin has been studied most extensively in septic shock, those findings cannot be directly extrapolated to postcardiac surgery vasoplegia. Septic shock is driven by infection-related inflammatory and microcirculatory dysfunction, whereas post-CPB vasoplegia occurs in the setting of surgical inflammation, endothelial injury, myocardial vulnerability, and variable ventricular reserve. Therefore, vasopressor selection in postcardiac surgery vasoplegia should account for perioperative hemodynamics, ventricular function, and operative context [7,20,21].

Interpretation of Included Studies

The randomized VANCS trial provided important evidence specific to postcardiac surgery vasoplegia, demonstrating that vasopressin, as the initial vasopressor strategy, was associated with fewer postoperative complications than norepinephrine, including lower rates of acute kidney injury and atrial fibrillation, as well as shorter ICU and hospital lengths of stay [8]. These findings are biologically plausible. Vasopressin preferentially constricts efferent renal arterioles and may improve renal perfusion pressure, which could explain the reduction in kidney injury observed in the randomized trial [16]. In addition, avoidance of excessive catecholamine exposure may reduce adrenergic-mediated arrhythmogenesis and myocardial oxygen demand. Previous perioperative studies have similarly suggested that low-dose vasopressin may improve hemodynamic stability and reduce vasopressor requirements during cardiac surgery [18,19].

In contrast, the propensity score-matched cohort study, including patients with severe preoperative left ventricular dysfunction, reported different clinical patterns, including higher rates of arrhythmias and mortality in patients receiving vasopressin [12]. Several explanations may account for these divergent findings. Patients with impaired ventricular function may be particularly sensitive to increases in afterload induced by potent vasoconstrictors. Vasopressin exerts its vasoconstrictive effects primarily through V1 receptor activation on vascular smooth muscle cells, leading to increased intracellular calcium and systemic vasoconstriction without direct inotropic support [22]. In a failing ventricle operating on a steep pressure-volume curve, this increase in afterload may reduce stroke volume and worsen cardiac output, without the compensatory β1-adrenergic inotropic effect provided by norepinephrine. Consistent with this mechanism, the observational cohort reported significantly higher vasoactive-inotropic scores and greater requirements for additional inotropic agents in the vasopressin group, suggesting vasopressin-induced afterload increase required pharmacologic compensation rather than hemodynamic recovery [12]. Additionally, observational studies remain vulnerable to residual confounding despite propensity score matching. In clinical practice, vasopressin is often reserved for patients with more severe or catecholamine-resistant vasoplegia, which may bias outcomes toward worse prognoses in nonrandomized analyses.

Clinical Implications

These contrasting findings raise the possibility that baseline ventricular function may influence response to vasopressor therapy; however, available evidence is insufficient to confirm effect modification, and this interpretation should be considered hypothesis-generating. In patients with preserved ventricular function and low systemic vascular resistance, vasopressin may offer catecholamine-sparing vasoconstriction. In patients with severe left ventricular dysfunction, however, vasopressin-induced afterload augmentation may be less well tolerated unless accompanied by adequate inotropic support and close assessment of cardiac output. Future studies should stratify patients by ventricular function, pulmonary vascular load, and shock phenotype [8,12].

Our review also underscores the limited quantity and low certainty of comparative evidence available in this area. Despite the clinical importance of vasoplegic shock after cardiac surgery, few studies directly compare vasopressin with norepinephrine as initial therapy. Differences in study design, patient populations, definitions of vasoplegia, and outcome measures further limit quantitative synthesis. As reflected in the GRADE assessment framework, the overall certainty of evidence for most outcomes remains low to very low because of risk of bias, inconsistency between studies, indirectness of populations, and imprecision (Appendix D) [23]. Future studies should focus on phenotype-driven vasopressor strategies in postcardiac surgery vasoplegia, including stratification by baseline ventricular function, shock severity, renal risk, and inflammatory burden. Ongoing and future randomized trials comparing vasopressin-based versus norepinephrine-based strategies in cardiac surgery populations may help clarify whether early vasopressin improves renal or composite postoperative outcomes, and whether treatment effects differ by ventricular function or hemodynamic phenotype.

Strengths and Limitations of the Review

This review has limitations. First, the small number of eligible studies prevented quantitative meta-analysis. Second, heterogeneity in patient populations, particularly with respect to baseline ventricular function and shock severity, may have influenced observed treatment effects. Third, one included study was observational and therefore subject to residual confounding despite propensity score matching. Fourth, variations in definitions of vasoplegia and postoperative complications may limit comparability across studies. This was particularly relevant for renal outcomes because VANCS reported acute kidney injury requiring dialysis, whereas Cheng et al. reported severe acute kidney injury using AKIN stage II/III criteria. Similarly, although broader vasodilatory shock terms were used in the search strategy to maximize sensitivity, eligibility was restricted to postcardiac surgery vasoplegic shock after CPB; therefore, evidence from broader vasodilatory shock populations should be considered indirect. Risk of bias was assessed using validated tools, including RoB 2 [10] for randomized trials and ROBINS-I [11] for observational studies, and the review was conducted in accordance with PRISMA [9] reporting standards.

## Conclusions

In patients with vasoplegic shock following cardiac surgery, current evidence is insufficient to establish either vasopressin or norepinephrine as definitively superior initial therapy. Although vasopressin may reduce postoperative complications in patients with preserved ventricular function, contrasting data in severe LV dysfunction suggest that treatment response may vary by clinical phenotype; this remains hypothesis-generating. Until higher-quality comparative data are available, vasopressor selection should be individualized according to ventricular function, shock phenotype, and renal risk. Adequately powered randomized trials stratified by these factors are needed to define the optimal first-line strategy.

## Appendices

Appendix A

**Table 4:** PRISMA Checklist

Section and Topic	Item #	Checklist Item	Location Where Item Is Reported
TITLE			
Title	1	Identify the report as a systematic review.	Title page (p. 1).
ABSTRACT			
Abstract	2	See the PRISMA 2020 for Abstracts checklist.	Abstract (pp. 3-4).
INTRODUCTION			
Rationale	3	Describe the rationale for the review in the context of existing knowledge.	Introduction (pp. 5-6).
Objectives	4	Provide an explicit statement of the objective(s) or question(s) the review addresses.	Introduction, final paragraph (p. 6).
METHODS			
Eligibility criteria	5	Specify the inclusion and exclusion criteria for the review and how studies were grouped for the syntheses.	Methods: Eligibility Criteria/Outcomes (pp. 7-8); Data Synthesis (pp. 9-10).
Information sources	6	Specify all databases, registers, websites, organisations, reference lists and other sources searched or consulted to identify studies. Specify the date when each source was last searched or consulted.	Methods: Literature Search (pp. 8-9); Suppl. Appendix 1 (pp. 1-2).
Search strategy	7	Present the full search strategies for all databases, registers and websites, including any filters and limits used.	Suppl. Appendix 1: complete search strategies (pp. 1-2).
Selection process	8	Specify the methods used to decide whether a study met the inclusion criteria of the review, including how many reviewers screened each record and each report retrieved, whether they worked independently, and if applicable, details of automation tools used in the process.	Methods: Study Selection (p. 9); Figure 1 (p. 24).
Data collection process	9	Specify the methods used to collect data from reports, including how many reviewers collected data from each report, whether they worked independently, any processes for obtaining or confirming data from study investigators, and if applicable, details of automation tools used in the process.	Methods: Data Extraction (p. 9).
Data items	10a	List and define all outcomes for which data were sought. Specify whether all results that were compatible with each outcome domain in each study were sought (e.g. for all measures, time points, analyses), and if not, the methods used to decide which results to collect.	Methods: Outcomes (p. 8); Tables 2-3 (pp. 22-23).
	10b	List and define all other variables for which data were sought (e.g. participant and intervention characteristics, funding sources). Describe any assumptions made about any missing or unclear information.	Methods: Data Extraction (p. 9); Table 1 (p. 21).
Study risk of bias assessment	11	Specify the methods used to assess risk of bias in the included studies, including details of the tool(s) used, how many reviewers assessed each study and whether they worked independently, and if applicable, details of automation tools used in the process.	Methods: Risk of Bias Assessment (p. 9); Suppl. Table 1.
Effect measures	12	Specify for each outcome the effect measure(s) (e.g. risk ratio, mean difference) used in the synthesis or presentation of results.	Tables 2-3 (pp. 22-23); effect measures as reported by original studies.
Synthesis methods	13a	Describe the processes used to decide which studies were eligible for each synthesis (e.g. tabulating the study intervention characteristics and comparing against the planned groups for each synthesis (item #5)).	Methods: Eligibility Criteria, Outcomes, and Data Synthesis (pp. 7-10).
	13b	Describe any methods required to prepare the data for presentation or synthesis, such as handling of missing summary statistics, or data conversions.	N/A-no meta-analysis or data conversions; Methods: Data Synthesis (pp. 9-10).
	13c	Describe any methods used to tabulate or visually display results of individual studies and syntheses.	Tables 1-3 (pp. 21-23); Figure 1 (p. 24).
	13d	Describe any methods used to synthesize results and provide a rationale for the choice(s). If meta-analysis was performed, describe the model(s), method(s) to identify the presence and extent of statistical heterogeneity, and software package(s) used.	Methods: Data Synthesis (pp. 9-10); narrative synthesis only.
	13e	Describe any methods used to explore possible causes of heterogeneity among study results (e.g. subgroup analysis, meta-regression).	Discussion: ventricular function/shock phenotype heterogeneity (pp. 14-18).
	13f	Describe any sensitivity analyses conducted to assess robustness of the synthesized results.	N/A-no quantitative synthesis; no sensitivity analysis performed.
Reporting bias assessment	14	Describe any methods used to assess risk of bias due to missing results in a synthesis (arising from reporting biases).	Suppl. Table 3; not formally assessed because only 2 studies were included.
Certainty assessment	15	Describe any methods used to assess certainty (or confidence) in the body of evidence for an outcome.	Suppl. Tables 2-3: GRADE certainty assessment.
RESULTS			
Study selection	16a	Describe the results of the search and selection process, from the number of records identified in the search to the number of studies included in the review, ideally using a flow diagram.	Results: Study Selection (p. 10); Figure 1 (p. 24).
	16b	Cite studies that might appear to meet the inclusion criteria, but which were excluded, and explain why they were excluded.	Results: Study Selection (p. 10); Figure 1 lists full-text exclusions (p. 24).
Study characteristics	17	Cite each included study and present its characteristics.	Results: Study Characteristics (pp. 10-11); Table 1 (p. 21).
Risk of bias in studies	18	Present assessments of risk of bias for each included study.	Results: Risk of Bias (pp. 13-14); Suppl. Table 1.
Results of individual studies	19	For all outcomes, present, for each study: (a) summary statistics for each group (where appropriate) and (b) an effect estimate and its precision (e.g. confidence/credible interval), ideally using structured tables or plots.	Results: Primary/Secondary Outcomes (pp. 11-13); Tables 2-3 (pp. 22-23).
Results of syntheses	20a	For each synthesis, briefly summarise the characteristics and risk of bias among contributing studies.	Results: Study Characteristics/Risk of Bias (pp. 10-14); Suppl. Table 1.
	20b	Present results of all statistical syntheses conducted. If meta-analysis was done, present for each the summary estimate and its precision (e.g. confidence/credible interval) and measures of statistical heterogeneity. If comparing groups, describe the direction of the effect.	N/A-no statistical synthesis/meta-analysis; narrative results in pp. 11-13 and Tables 2-3.
	20c	Present results of all investigations of possible causes of heterogeneity among study results.	Discussion (pp. 14-18); no formal subgroup analysis/meta-regression.
	20d	Present results of all sensitivity analyses conducted to assess the robustness of the synthesized results.	N/A-no sensitivity analyses conducted.
Reporting biases	21	Present assessments of risk of bias due to missing results (arising from reporting biases) for each synthesis assessed.	Suppl. Table 3; publication bias could not be formally assessed.
Certainty of evidence	22	Present assessments of certainty (or confidence) in the body of evidence for each outcome assessed.	Suppl. Tables 2-3; Discussion (p. 18).
DISCUSSION			
Discussion	23a	Provide a general interpretation of the results in the context of other evidence.	Discussion (pp. 14-18).
	23b	Discuss any limitations of the evidence included in the review.	Discussion: limitations paragraph (pp. 18-19).
	23c	Discuss any limitations of the review processes used.	Discussion: limitations paragraph (pp. 18-19).
	23d	Discuss implications of the results for practice, policy, and future research.	Discussion (pp. 17-19); Conclusion (p. 19).
OTHER INFORMATION			
Registration and protocol	24a	Provide registration information for the review, including register name and registration number, or state that the review was not registered.	Methods: Study Design and Protocol Registration (p. 7).
	24b	Indicate where the review protocol can be accessed, or state that a protocol was not prepared.	Methods: Protocol Registration (p. 7); Suppl. Appendix 1 includes PROSPERO link.
	24c	Describe and explain any amendments to information provided at registration or in the protocol.	No protocol amendments reported.
Support	25	Describe sources of financial or non-financial support for the review, and the role of the funders or sponsors in the review.	Title page: Funding statement (p. 2).
Competing interests	26	Declare any competing interests of review authors.	Title page: Competing interest statement (p. 2).
Availability of data, code and other materials	27	Report which of the following are publicly available and where they can be found: template data collection forms; data extracted from included studies; data used for all analyses; analytic code; any other materials used in the review.	Title page: Data availability statement (p. 2); Suppl. Appendix/Tables.

Appendix B

Full Boolean Search Strategies

The full search strategy was prospectively registered and is available via the PROSPERO record (CRD420261341741) (https://www.crd.york.ac.uk/PROSPEROFILES/e6b99dae7b6349665db222b931b1fd04.pdf).

Database 1: MEDLINE (via PubMed)

Searched: From inception to March 2026. No language or date restrictions.

("vasoplegic syndrome"[MeSH Terms] OR "vasoplegia"[tiab] OR "vasoplegic shock"[tiab] 

OR "vasodilatory shock"[tiab] OR "distributive shock"[tiab] OR "vasoplegic syndrome"[tiab])

AND

("cardiac surgical procedures"[MeSH Terms] OR "cardiac surgery"[tiab] OR "heart surgery"[tiab] 

OR "cardiopulmonary bypass"[MeSH Terms] OR "cardiopulmonary bypass"[tiab] 

OR "extracorporeal circulation"[tiab] OR "coronary artery bypass"[MeSH Terms] 

OR "valve surgery"[tiab] OR "aortic surgery"[tiab])

AND

("vasopressins"[MeSH Terms] OR "vasopressin"[tiab] OR "arginine vasopressin"[tiab] 

OR "AVP"[tiab] OR "antidiuretic hormone"[tiab] OR "pitressin"[tiab])

AND

("norepinephrine"[MeSH Terms] OR "norepinephrine"[tiab] OR "noradrenaline"[tiab])

Database 2: Embase (via Ovid)

Searched: inception - March 2026. No language or date restrictions.

(vasoplegia/ OR vasoplegic shock/ OR vasodilatory shock/ OR distributive shock/ 

OR vasoplegia.ti,ab OR vasoplegic shock.ti,ab OR vasodilatory shock.ti,ab 

OR vasoplegic syndrome.ti,ab)

AND

(heart surgery/ OR cardiac surgery.ti,ab OR heart surgery.ti,ab 

OR cardiopulmonary bypass/ OR cardiopulmonary bypass.ti,ab 

OR extracorporeal circulation/ OR extracorporeal circulation.ti,ab 

OR coronary artery bypass graft/ OR valve surgery.ti,ab OR aortic surgery.ti,ab)

AND

(vasopressin/ OR vasopressin.ti,ab OR arginine vasopressin.ti,ab 

OR AVP.ti,ab OR antidiuretic hormone.ti,ab OR pitressin.ti,ab)

AND

(noradrenalin/ OR norepinephrine.ti,ab OR noradrenaline.ti,ab)

Database 3: Cochrane Central Register of Controlled Trials (CENTRAL)

Searched via Cochrane Library: inception - March 2026. No language or date restrictions.

(vasoplegia OR "vasoplegic shock" OR "vasodilatory shock" OR "vasoplegic syndrome" 

OR "distributive shock")

AND

("cardiac surgery" OR "heart surgery" OR "cardiopulmonary bypass" 

OR "extracorporeal circulation" OR "coronary artery bypass" 

OR "valve surgery" OR "aortic surgery")

AND

(vasopressin OR "arginine vasopressin" OR AVP OR pitressin)

AND

(norepinephrine OR noradrenaline)

Database 4: Science Citation Index (Web of Science)

Searched: inception - March 2026. No language or date restrictions.

TS=(vasoplegia OR "vasoplegic shock" OR "vasodilatory shock" OR "vasoplegic syndrome")

AND

TS=("cardiac surgery" OR "heart surgery" OR "cardiopulmonary bypass" 

OR "extracorporeal circulation" OR "coronary artery bypass" OR "valve surgery")

AND

TS=(vasopressin OR "arginine vasopressin" OR AVP OR pitressin)

AND

TS=(norepinephrine OR noradrenaline)

Database 5: Scopus

Searched: inception - March 2026. No language or date restrictions.

TITLE-ABS-KEY(vasoplegia OR "vasoplegic shock" OR "vasodilatory shock" 

OR "vasoplegic syndrome" OR "distributive shock")

AND

TITLE-ABS-KEY("cardiac surgery" OR "heart surgery" OR "cardiopulmonary bypass" 

OR "extracorporeal circulation" OR "coronary artery bypass" OR "valve surgery")

AND

TITLE-ABS-KEY(vasopressin OR "arginine vasopressin" OR AVP OR pitressin)

AND

TITLE-ABS-KEY(norepinephrine OR noradrenaline)

Trial Registries

Searched: March 2026

ClinicalTrials.gov (clinicaltrials.gov): Search terms: "vasoplegia" AND "vasopressin"; "vasoplegic shock" AND "cardiac surgery"

WHO ICTRP (apps.who.int/trialsearch): Search terms: "vasoplegia vasopressin cardiac surgery"

Appendix C

**Table 5:** Risk of Bias Assessment of Included Studies Notes: Risk of bias for the randomized trial was assessed using the Cochrane RoB 2 tool, and the observational cohort was evaluated using the ROBINS-I tool. RoB 2: Cochrane Risk of Bias tool for randomized trials; ROBINS-I: Risk Of Bias In Non-randomized Studies of Interventions

Domain	Hajjar et al., 2017 (VANCS) [8]	Cheng et al., 2018 [12]
Study design	Randomized controlled trial	Observational cohort (propensity score–matched)
Randomization process	Low risk	Not applicable
Allocation concealment	Low risk	Not applicable
Blinding of participants and personnel	Low risk	High risk
Outcome assessment	Low risk	Moderate risk
Confounding	Low risk	Serious risk
Selection of participants	Low risk	Moderate risk
Overall risk of bias	Some concerns	Serious risk

Appendix D 

**Table 6:** GRADE Assessment of Certainty of Evidence for Key Outcomes GRADE definitions: High = very confident that the true effect lies close to the estimate. Moderate = moderate confidence in the effect estimate; the true effect is likely close but may differ substantially. Low = limited confidence in the estimate; the true effect may be substantially different. Very low = very little confidence in the estimate; the true effect is likely substantially different.

Outcome	Certainty of Evidence	Reasons for Downgrading
Acute kidney injury	Low	Inconsistency (opposite effects across studies), indirectness (different AKI definitions and patient populations), imprecision (limited number of studies and events)
Atrial fibrillation	Low	Inconsistency (vasopressin beneficial in the VANCS trial but harmful in the Cheng cohort), indirectness (differences in baseline ventricular function between study populations)
Mortality	Very low	Risk of bias (serious bias in the observational study), inconsistency between studies, imprecision (few events), indirectness
ICU length of stay	Very low	Inconsistency (opposite direction of effect between studies), imprecision (median values and limited sample size), risk of bias, indirectness

Appendix E

**Table 7:** GRADE Domain Assessment Across Included Studies Notes: Domains were assessed according to the GRADE framework, considering study design, consistency of results, directness of evidence, precision of estimates, and potential publication bias. GRADE: Grading of Recommendations Assessment, Development and Evaluation; LV: left ventricular

Domain	Hajjar et al., 2017 (RCT) [8]	Cheng et al., 2018 (Cohort) [12]	Overall Assessment
Risk of bias	Some concerns	Serious risk	Downgraded 1 level
Inconsistency	Benefit of vasopressin	Neutral or potentially harmful effect	Downgraded 1-2 levels
Indirectness	Preserved ventricular function population	Severe LV dysfunction population	Downgraded 1 level
Imprecision	Moderate sample size	Few events and wide confidence intervals	Downgraded 1 level
Publication bias	Cannot be formally assessed	Cannot be formally assessed	Not downgraded
